# Individualized Nomogram for Predicting Survival in Patients with Brain Metastases After Stereotactic Radiosurgery Utilizing Driver Gene Mutations and Volumetric Surrogates

**DOI:** 10.3389/fonc.2021.659538

**Published:** 2021-05-13

**Authors:** Cheng Zhou, Changguo Shan, Mingyao Lai, Zhaoming Zhou, Junjie Zhen, Guanhua Deng, Hainan Li, Juan Li, Chen Ren, Jian Wang, Ming Lu, Liang Zhang, Taihua Wu, Dan Zhu, Feng-Ming (Spring) Kong, Longhua Chen, Linbo Cai, Lei Wen

**Affiliations:** ^1^ Department of Oncology, Guangdong Sanjiu Brain Hospital, Guangzhou, China; ^2^ Department of Radiation Medicine, School of Public Health, Southern Medical University, Guangzhou, China; ^3^ Department of Pathology, Guangdong Sanjiu Brain Hospital, Guangzhou, China; ^4^ Department of Radiation Oncology, Nanfang Hospital, Southern Medical University, Guangzhou, China; ^5^ Department of Neurosurgery, Guangdong Sanjiu Brain Hospital, Guangzhou, China; ^6^ Department of Clinical Oncology, The University of Hong Kong Shenzhen Hospital, Shenzhen, China

**Keywords:** brain metastases, stereotactic radiosurgery, nomogram, gene mutation, prediction model

## Abstract

It is well-known that genomic mutational analysis plays a significant role in patients with NSCLC for personalized treatment. Given the increasing use of stereotactic radiosurgery (SRS) for brain metastases (BM), there is an emerging need for more precise assessment of survival outcomes after SRS. Patients with BM and treated by SRS were eligible in this study. The primary endpoint was overall survival (OS). Cox regression models were used to identify independent prognostic factors. A survival predictive nomogram was developed and evaluated by Concordance-index (C-index), area under the curve (AUC), and calibration curve. From January 2016 to December 2019, a total of 356 BM patients were eligible. The median OS was 17.7 months [95% confidence interval (CI) 15.5–19.9] and the actual OS at 1- and 2-years measured 63.2 and 37.6%, respectively. A nomogram for OS was developed by incorporating four independent prognostic factors: Karnofsky Performance Score, cumulative tumor volume, gene mutation status, and serum lactate dehydrogenase. The nomogram was validated in a separate cohort and demonstrated good calibration and good discriminative ability (C-index = 0.780, AUC = 0.784). The prognostic accuracy of the nomogram (0.792) was considerably enhanced when compared with classical prognostic indices, including the Graded Prognostic Assessment (0.708), recursive partitioning analysis (0.587), and the SRS (0.536). Kaplan–Meier curves showed significant differences in OS among the stratified low-, median- and high-risk groups (*P* < 0.001). In conclusion, we developed and validated an individualized prognostic nomogram by integrating physiological, volumetric, clinical chemistry, and molecular biological surrogates. Although this nomogram should be validated by independent external study, it has a potential to facilitate more precise risk-stratifications to guide personalized treatment for BM.

## Introduction

Brain metastases (BM) represent the most common intracranial tumors in adults, which occur up to 10-times more frequently than primary central nervous system tumors. For brain metastases, stereotactic radiosurgery (SRS) offers an excellent minimally invasive ablative treatment option due to its favorable local control efficacy and late onset toxicity (1, 2). Previous reports have indicated that the survival probability for patients after SRS varies with age, Karnofsky Performance Status (KPS), primary cancer site, driver gene mutations, tumor volume, number of metastatic sites, extracranial disease burden, and systemic treatments (3). Nevertheless, the prognosis of BM is rather complex, and the weighted value for a variety of risk factors in the prediction of survival outcomes is largely unknown.

Several well-known prognostic indices are widely utilized for clinical decision-making and outcome research, and include recursive partitioning analysis (RPA), the Score Index for Radiosurgery (SIR), the Basic Score for Brain Metastases (BSBM), and the Graded Prognostic Assessment (GPA) (4–6). These prognostic scoring systems were established by integrating several clinicopathological features such as age, the KPS, the number of metastatic lesions, extracranial metastases, and the control of the primary tumor, and allowed a certain degree of prognostic discrimination. In the era of precision medicine, the identification of driver gene mutations is essential to understand the molecular profiles of tumors and hence provides specific insights for risk assessment as well as tailored treatment (7). Furthermore, volumetric and clinical chemistry parameters might also be associated with prognosis (8, 9). In the light of new knowledge in cancer biology, the incorporation of molecular and physiological tumor characteristics into clinical stratification schemes may further advance the prognostic predictive capacity for brain metastases patients who received SRS.

The nomogram is widely used to predict specific prognosis of cancer patients in the form of numerical probability by quantifying each prognostic factor (10, 11). The present study aimed to identify independent prognostic factors using a large retrospective cohort of patients with brain metastases. Given the important insight from currently available prognostic indices (4–6), we will further develop and validate a multivariable nomogram prediction model by integrating several featured molecular and physiological surrogates. The established prognostic algorithm could thus facilitate personalized surveillance programs and appropriate treatment strategies for this devastating disease following SRS.

## Methods

### Patient Population and Data Collection

Between 1 Jan 2016 and 31 December 2019, a total of 594 patients with brain metastases extracted from a prospectively compiled database at our institution were screened. This study was conducted under the Institutional Ethics Committee approved retrospective review, which included a waiver for the requirement of informed consent for participation in the study. Patients were eligible if they: a) had a pathologically proven primary cancer; and b) had undergone SRS for a newly diagnosed BM. The exclusion criteria were: a) tumor combined with leptomeningeal metastases, b) diffuse or countless metastases ineligible for SRS, and c) surgical resection of metastatic lesions before SRS. A total of 356 patients were finally included in the present study. For the nomogram analysis, patients were randomly divided into a training set (n = 230) and a validation set (n = 126) using a random number generator by R software (**Supplementary Figure S1**). Detailed patient characteristics were collected. We evaluated all brain metastatic lesions based on contrast-enhanced MRI. Largest tumor volume was defined as the largest contiguous lesion present on the pre-SRS (T1-weighted postcontrast image). The cumulative tumor volume (CTV) was defined as the sum of tumor volume of all treated BM lesions. For example, a female patient with two metastatic lesions in the brain, the diameter and volume of the two lesions were 4.3 cm and 14.43 cm^3^, 1.7 cm and 1.47 cm^3^, respectively (**Supplementary Figure S2**). Then diameter of the largest tumor was 4.3 cm, the largest tumor volume was 14.43 cm^3^, and CTV was 15.90 cm^3^ (14.43 cm^3^ plus 1.47 cm^3^).

### Stereotactic Radiosurgery Treatment

All patients included were treated by single or fractionated SRS (FSRS) *via* the Novalis Tx^®^ system (BrainLAB AG, Feldkirchen, Germany; Varian Medical System, Palo Alto, CA, USA). In brief, patients were treated either by single SRS with the radiation dose of 16–18 Gy, or FSRS in two or three fractions at 8–12 Gy/fraction. For FSRS, fractions were administrated with an interval of 1–3 days. Prophylactic dehydration measures such as mannitol were regularly administrated after SRS unless there were contraindications.

### Statistics and Nomogram Development

The endpoint of the present study was overall survival (OS), which was defined as the time from SRS treatment to death from any cause or censored at the date of last follow-up unless otherwise specified. Descriptive statistics for quantitative variables were expressed as means (± standard deviation, SD) or medians (interquartile range, IQR), and categorical variables were expressed as numbers (percentages). OS was estimated using Kaplan–Meier analysis.

The American Joint Committee on Cancer (AJCC)-proposed checklist was used for guidance in building the prediction model (12). In the training cohort, Cox proportional hazards models were used to identify significant prognostic factors associated with OS. A stepwise variable selection with *P-*values less than 0.15 by univariable analysis was used as the criteria for entry and retention in the multivariable analysis. Hazard ratios (HRs) were presented with their 95% confidence intervals (CIs). Continuous predictors [*i.e.*, tumor size, largest tumor volume (LTV), and CTV] were categorized by optimal cutoffs using the receiver-operating characteristic (ROC) curve method with 1-year OS as the dependent variable and tumor diameter, LTV, and CTV as the independent variables (13). The optimal cutoffs of tumor diameter, LTV, and CTV that maximized sensitivity while minimizing 1-specificity were determined to be 2.0 cm, 2.5 cm^3^, and 3.5 cm^3^, respectively. The KPS score was calculated as a continuous variable, while age (≥65 *vs <*65) and serum lactate dehydrogenase (LDH) (<200 *vs* 200–300 *vs >*300) as categorical variables in Cox regression analysis. Cox proportional hazards models were utilized to predicting clinical outcomes in the constructed nomogram for 1-year and 2-year OS rates for patients with brain metastases after SRS.

The established nomogram was further analyzed in a validation cohort. Model performance was assessed by the predictive accuracy (discriminating ability) and by the accuracy of point estimates of the survival function (calibration). The value of the Concordance index (C-index) and the area under the curve (AUC) were used to evaluate the discriminative ability of the nomogram (14). A C-index of 0.5 indicates the absence of discrimination, whereas 1.0 indicates perfect separation of patients with different outcomes. Calibration was evaluated using a calibration plot to compare the relationship between the observed outcome frequencies *versus* the predicted outcomes.

For each patient, the total number of points based on the nomogram was calculated and the patients were stratified into three risk groups (high-, medium-, and low-) based on the sum of the points, the 25^th^ and 75^th^ percentiles of the sum of risk scores were used as the cutoff values (15). Kaplan–Meier curves of the three risk group patients were plotted to further assess calibration. The prediction capacity of the established nomogram was also compared with the more well-established GPA, RPA, and SIR models by ROC curves. All analyses were performed using R version 4.0.2 (https://www.rproject.org), SPSS version 26 (IBM, Armonk, NY, USA) and GraphPad Prism 6.0 (San Diego, California, USA). All statistical tests were two sided, and *P-*value <0.05 was considered statistically significant.

## Results

### Patient Characteristics and Survival

A total of 356 patients who received SRS for 1,481 brain metastases were analyzed. The median follow-up was 12.2 months (range 1.5–34.1 months) for living patients. A summary of the patient demographics and tumor characteristics is shown in **Table 1**. Most patients (268/356, 75.3%) were diagnosed with a primary NSCLC, followed by breast cancer (38/356, 10.7%), digestive system cancer (23/356, 6.5%), and other cancer types (27/356, 7.6%). Among the 268 BM patients with NSCLC, 146 harbored an *EGFR/ALK* mutation and 122 were *EGFR/ALK* wild-type or of unknown gene status. Most patients (72.3%) had multiple BM lesions and the median CTV was 9.5 cm^3^ (IQR 2.3–21.5). Single SRS was conducted in 189 patients while FSRS (2–3 fx) in 167 patients. The median biologically effective dose (BED) of radiation was 41.6 Gy (IQR 41.6–50.4) for the *α/β* value of 10 (16). In the overall cohort, the median OS was 17.7 months (95% CI 15.5–19.9). Actual 1- and 2-year OS rates were 63.2 and 37.6%, respectively.

**Table 1:** Demographics and clinical characteristics of the study population.

Characteristics	Overall cohort	Training Cohort	Validation Cohort
	N = 356	(n = 230)	(n = 126)
**Gender, n (%)**			
**Male**	188 (52.8%)	120 (52.2%)	68 (54.0%)
** Female**	168 (47.2%)	110 (47.8%)	58 (46.0%)
**Age (yrs.)**			
** Median (IQR)**	58 (49–65)	58 (49–65)	57 (49–64)
** ≥65**	92 (25.8%)	61 (26.5%)	31 (24.6%)
** <65**	264 (74.2%)	169 (73.5%)	95 (75.4%)
**KPS**			
**Median (IQR)**	80 (70–80)	80 (70–80)	80 (70–90)
** ≥70**	280 (78.7%)	181 (78.7%)	99 (78.6%)
** <70**	76 (21.3%)	49 (21.3%)	27 (21.4%)
**Primary tumor**			
** NSCLC**	268 (75.3%)	170 (73.9%)	98 (77.8%)
** Breast cancer**	38 (10.7%)	26 (11.3%)	12 (9.5%)
** Digestive system cancer**	23 (6.5%)	15 (6.5%)	8 (6.3%)
** Others**	27 (7.6%)	19 (8.3%)	8 (6.3%)
**Mutation status**			
** NSCLC mutant**	146 (41.0%)	90 (39.1%)	56 (44.4%)
** NSCLC wild type/unknown**	122 (34.3%)	79 (34.3%)	43 (34.1%)
** N.A. (non-NSCLC)**	88 (24.7%)	61 (26.5%)	27 (21.4%)
**Systemic disease control**			
** Controlled**	146 (41.0%)	97 (42.2%)	50 (39.7%)
** Uncontrolled**	210 (59.0%)	134 (58.3%)	76 (60.3%)
** Number of BM**			
** Solitary**	97 (27.2%)	65 (28.3%)	32 (25.4%)
** Multiple**	259 (72.8%)	165 (71.7%)	94 (74.6%)
**Distribution of BM**			
** Supratentorial**	166 (46.6%)	108 (47.0%)	58 (46.0%)
** Infratentorial**	34 (9.6%)	21 (9.1%)	13 (10.3%)
** Both**	156 (43.8%)	99 (43.0%)	57 (45.2%)
**Diameter of largest tumor (cm)**			
** Median (IQR)**	2.7 (1.7–3.9)	2.7 (1.8–3.9)	2.7 (1.7–3.9)
** ≥2.5**	197 (55.3%)	128 (55.7%)	69 (54.8%)
**<2.5**	159 (44.7%)	102 (44.3%)	57 (45.2%)
**Largest tumor volume (cm^3^)**			
** Median (IQR)**	6.2 (1.6–15.4)	6.3 (1.7–15.5)	6.1 (1.6–15.3)
** ≥2.5**	241 (67.7%)	156 (67.8%)	85 (67.5%)
**<2.5**	115 (32.3%)	74 (32.2%)	41 (32.5%)
**Cumulative tumor volume (cm^3^)**			
** Median (IQR)**	9.5 (2.3–21.5)	9.5 (2.4–21.0)	9.5 (2.3–22.2)
** ≥3.5**	242 (68.0%)	156 (67.8%)	86 (68.3%)
**<3.5**	114 (32.0%)	74 (32.2%)	40 (31.7%)
**SRS/FSRS**			
** Single SRS**	189 (53.1%)	125 (54.3%)	64 (50.8%)
** FSRS**	167 (46.9%)	105 (45.7%)	62 (49.2%)
**BED (Gy)**			
**Median (IQR)**	41.6 (41.6–50.4)	43.2 (41.6–50.4)	41.6 (41.6–50.4)
**LDH (U/L)**			
** Median (IQR)**	197 (167–249)	197 (167–247)	201 (167–257)
** <200**	181 (50.8%)	119 (31.3%)	62 (49.2%)
** 200–300**	122 (34.3%)	77 (22.6%)	45 (35.7%)
**>300**	53 (14.9%)	34 (9.6%)	19 (15.1%)

KPS, Karnofsky Performance Score; N.A., not applicable; NSCLC, non-small cell lung cancer; BM, brain metastases; IQR, interquartile range; SRS, stereotactic radiosurgery; FSRS, fractionated stereotactic radiosurgery; BED, biologically effective dose; LDH, lactate dehydrogenase.

### Univariate and Multivariate Analyses for Overall Survival in the Training Cohort

**Figure 1:**
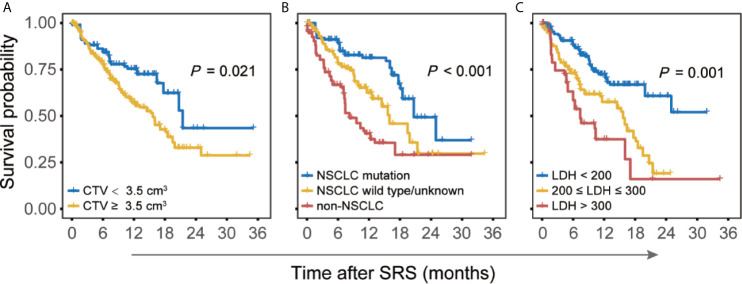
Kaplan–Meier curves for overall survival after stereotactic radiosurgery (SRS) in patients with brain metastases (BM) with reference to **(A)** cumulative tumor volume (CTV), **(B)** primary tumor type (mutation status), and **(C)** serum lactate dehydrogenase (LDH) levels.

Univariable and multivariable Cox regression analysis were performed to assess variables associated with the OS in training cohort (**Table 2**). Univariable analysis identified several significant variables for OS: age, KPS score, mutation status, CTV, and serum LDH **(** **Figure 1** **)**. These five significant variables, together with three marginally significant factors (sex, systematic disease status, and largest tumor volume) were included in the multivariate analysis. According to multivariable analysis, the KPS score (*P* = 0.049), mutation status (*P* < 0.001), cumulative tumor volume (*P* = 0.021), as well as serum LDH (*P* = 0.001) were independently associated with OS in the training cohort.

**Table 2:** Univariate and multivariate analysis for overall survival in BM patients treated by SRS in the training cohort.

Covariate	Univariate analysis		Multivariate analysis	
	HR (95%CI)	*P-value*	HR (95%CI)	*P-value*
**Gender**				
** Male**	1 [Reference]		1 [Reference]	
** Female**	0.699 (0.452–1.081)	0.107	0.747 (0.473–1.179)	0.210
**Age**				
** <65**	1 [Reference]			
** ≥65**	1.192 (0.732–1.941)	0.480		
**KPS**	0.975 (0.960–0.991)	0.002	0.981 (0.962–1.000)	0.049
**Mutation status**				
** NSCLC mutant**	1 [Reference]		1 [Reference]	
** NSCLC wild type/unknown**	1.736 (0.993–3.036)	0.053	1.517 (0.813–2.832)	0.078
** N.A. (non-NSCLC)**	3.058 (1.734–5.393)	<0.001	2.984 (1.627–5.472)	<0.001
**Systemic disease status**				
** Controlled**	1 [Reference]		1 [Reference]	
** Uncontrolled**	1.446 (0.909–2.299)	0.120	1.273 (0.781–2.075)	0.333
**Number of BM**				
** Solitary**	1 [Reference]			
** Multiple**	0.964 (0.586–1.587)	0.887		
**Distribution of BM**				
** Supratentorial**	1 [Reference]			
** Infratentorial**	0.866 (0.401–1.869)	0.714		
** Both**	0.943 (0.587–1.515)	0.809		
**Location of largest tumor**				
** Supratentorial**	1 [Reference]			
** Infratentorial**	1.108 (0.672–1.828)	0.688		
**Diameter of largest tumor (cm)**				
** <2.5**	1 [Reference]		1 [Reference]	
** ≥2.5**	1.650 (0.988–2.755)	0.055	1.714 (0.833–3.530)	0.144
**Largest tumor volume (cm^3^)**				
** <2.5**	1 [Reference]		1 [Reference]	
** ≥2.5**	1.449 (0.890–2.357)	0.136	1.819 (0.575–5.747)	0.308
**Cumulative tumor volume (cm^3^)**				
** <3.5**	1 [Reference]		1 [Reference]	
** ≥3.5**	1.758 (1.063–2.907)	0.028	3.369 (1.109–10.232)	0.032
**LDH**				
** <200**	1 [Reference]		1 [Reference]	
** 200–300**	2.144 (1.319–3.487)	0.002	1.852 (1.109–3.095)	0.005
** >300**	3.124 (1.734–5.628)	>0.001	2.640 (1.390–5.011)	0.001
**SRS**				
**Single SRS**	1 [Reference]			
** FSRS**	1.361 (0.883–2.099)	0.163		
**BED**				
** <43.2**	1 [Reference]			
** ≥43.2**	0.942 (0.601–1.477)	0.796		

HR, hazard ratio; CI, confidence interval; KPS, Karnofsky Performance Score; N.A., not applicable; NSCLC, non-small cell lung cancer; BM, brain metastases; SRS, stereotactic radiosurgery; FSRS, fractionated stereotactic radiosurgery; BED, biologically effective dose; LDH, lactate dehydrogenase. Underlined values: the P value of mutation status on OS.

### Development of a Nomogram for Overall Survival

**Figure 2:**
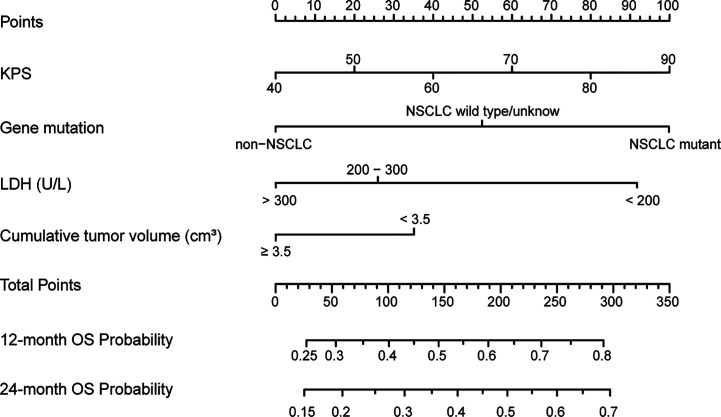
The established nomogram to predict overall survival created based on four independent prognostic factors.

Based on identified predictive factors from the training cohort, we developed a nomogram to predict the OS of the patients with brain metastases after SRS **(** **Figure 2** **)**. The nomogram integrated four factors: the KPS score (range 40–90), primary cancer and mutation status (NSCLC mutation or NSCLC wild-type/unknown or non-NSCLC), CTV (<3.5 or ≥3.5 cc) and serum LDH levels (<200 or 200–300 or >300 U/L). Higher total points based on the sum of the assigned number of points for each factor in the nomogram indicated a favorable OS. For example, a patient with a good KPS score (80 points), EGFR-mutant NSCLC, large CTV (5.2 cc) and medium serum LDH levels (270 U/L) would have a total of 205.5 points (80 points for KPS, 100 points for EGFR mutation, 25.5 points for LDH, and 0 points for CTV), for a predicted 1-year and 2-year OS of 63.5 and 50%, respectively.

### Nomogram Validation and Evaluation

**Figure 3:**
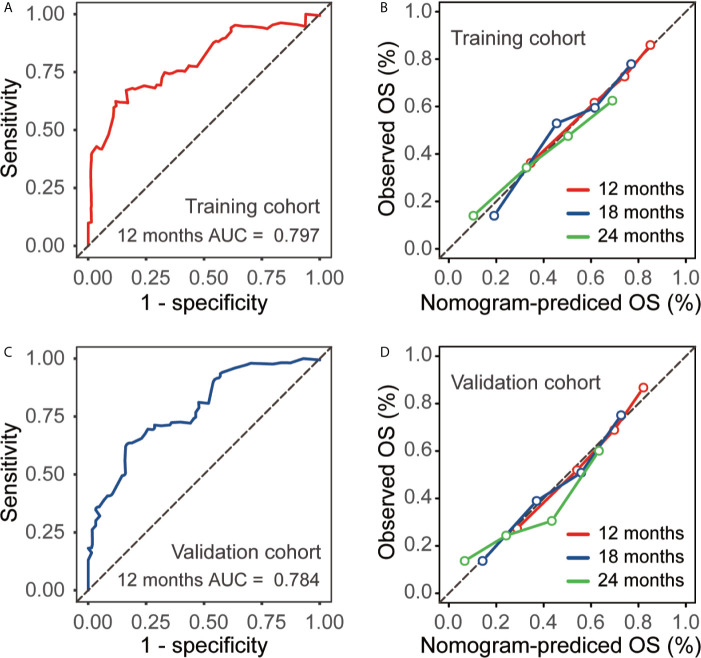
Receiver operating characteristic (ROC) analysis and calibration curves for the training and validation cohort. **(A)** ROC curve for the prediction model in the training cohort. **(B)** Calibration plot comparing nomogram-predicted and observed overall survival in the training cohort. **(C)** ROC for the prediction model in the validation cohort. **(D)** Calibration plot comparing nomogram-predicted and observed overall survival in the validation cohort.

The established nomogram was validated internally with a separate validation cohort. The C-index of nomogram to predict OS in the training cohort, validation cohort, and overall cohort were 0.792, 0.780, and 0.788, respectively. The AUC of the nomogram for the prediction of 12-month OS was 0.797 for the training cohort **(** **Figure 3A** **)**, 0.784 for the validation cohort **(** **Figure 3C** **)**, and 0.792 for the overall cohort. Furthermore, the calibration plots presented good agreement for the 12-, 18- and 24-month OS in the training and validation cohorts between the nomogram-predicted and actual observed OS rates **(** **Figures 3B, D** **)**.

**Figure 4:**
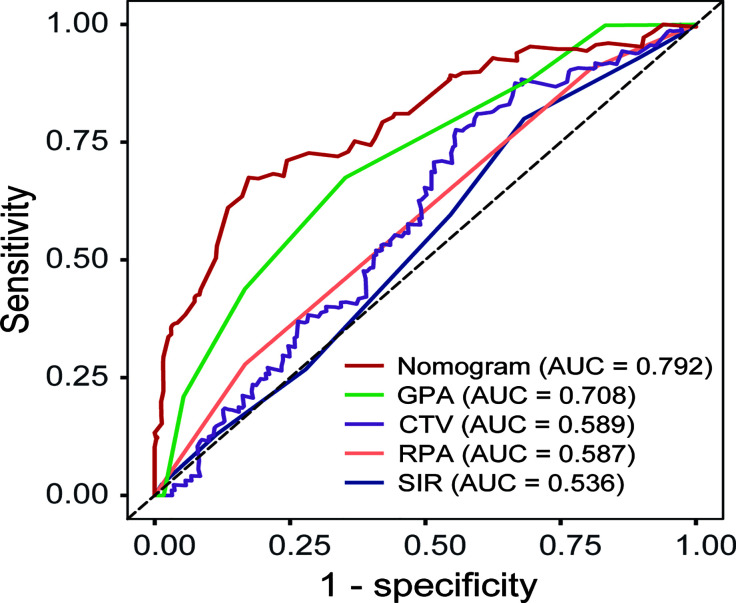
Receiver operating characteristic curve (ROC) comparing the predictive value of the present nomogram, GPA, RPA, SIR models, and cumulative tumor volume (CTV) alone for the prognosis of BM after SRS.

**Figure 5:**
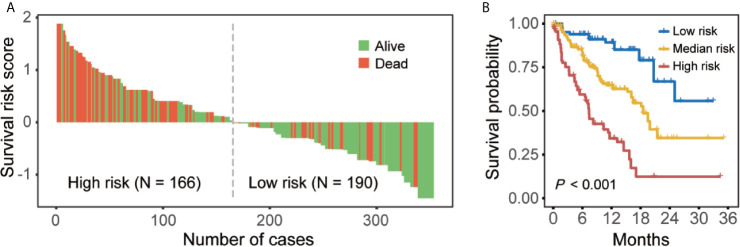
Nomogram-based risk stratifications for BM patients. **(A)** Waterfall plot of risk scores from nomogram prediction. **(B)** Kaplan–Meier curves for overall survival for patients with low-, medium-, and high-risk scores in the overall cohort.

The prediction values of this nomogram were compared with more well-established prognostic indices including the GPA, RPA, and SIR models as well as the volumetric variable CTV. In the overall cohort, the AUC of the nomogram (0.792) was higher than that of the GPA (0.708), CTV (0.589), RPA (0.587), and SIR (0.536) models **(** **Figure 4** **)**. According to nomogram predicted risk scores, patients from the overall cohort were classified into high-risk and low-risk groups. As a result, the distributions of the death events were predominant in the high-risk group compared to the low-risk group (**Figure 5A**). The Kaplan–Meier survival curve demonstrated a significant difference in OS among low-, median-, and high-risk groups with reference to the total risk score by the 25^th^ and 75^th^ percentiles (*P* < 0.001) (**Figure 5B**).

## Discussion

Given the increasingly recognized role of SRS in the treatment of brain metastases, specific scoring criteria integrating a spectrum of volumetric, physiological, clinical chemistry, and molecular biological surrogates for precise assessment of patients following SRS have yet to be established. Notably, the long-term survival of BM patients after SRS was not only associated with features of local BM lesions, but also with the outcome of post-SRS systematic treatment, *i.e.*, molecular characteristics of primary cancer. In the present study, we demonstrated that the cumulative tumor volume, driver gene mutation status, serum LDH, and KPS were significant prognostic factors for brain metastases after SRS. Furthermore, we developed and validated a robust nomogram to predict overall survival in patients with BM treated by SRS. By incorporating these four-independent prognostic clinicopathological parameters, the established nomogram exhibited excellent performance. It was found to have a robust AUC for the prediction of OS and enhanced prediction accuracy compared to classical prognostic indices.

Several well-known prognostic models had been established for BM. Based on analysis from three consecutive Radiation Therapy Oncology Group (RTOG) trials conducted between 1979 and 1993, the RTOG RPA divided patients into three distinct prognostic classes according to four clinical variables: age, KPS, controlled primary tumor, and extracranial metastases (4). Similar to the RPA, the new index, GPA, was developed from five randomized trials in 2008 (17). SIR was also a reliable prognostic score index for patients with BM submitted to SRS (18). A nomogram can calculate individualized estimates of prognosis and have been widely used. Using the nomogram proposed by Tien et al., the probability of survival with first-line paclitaxel and carboplatin with or without bevacizumab in non-squamous NSCLC patients can be estimated (19). Zhang F et al. (20) established an effective nomogram which could be used to identify high-risk patients of brain metastases after resection of primary lung cancer. Diandra NA et al. (21) and Daniel G et al. (22) had developed nomograms to predict distant brain failure and whole brain radiotherapy-free survival for brain metastases after SRS, respectively. To our knowledge, our proposed nomogram is among the first to predict OS for patients with BM following SRS.

Owing to the spatial limitation of the skull, clinical manifestations as well as prognosis is subjective to several volumetric factors such as the number, location, and volume of intracranial metastatic lesions. SIR is a well-established prognostic score specific for brain metastases patients treated by SRS (18). Compared to RPA and GPA, SIR assessment integrated the largest brain lesion volume, which is a critical factor for SRS. Our study comprehensively evaluated the impact of physical characteristics of brain metastases lesions on OS, and included the number of BM metastasis, distribution of metastases, location of the largest tumor, the diameter of the largest tumor, the largest tumor volume and CTV. Although the largest tumor volume or number of metastases were widely considered to affect long-term survival of brain metastases after SRS (6, 18), multivariate analysis in the present study revealed CTV played an overwhelming role when using OS as the endpoint. Our results are consistent with the serial studies from Chen et al. (23), whereby cumulative intracranial tumor volume was a superior prognostic factor compared to largest intracranial tumor volume in radiosurgery-treated BM patients. They also found that cumulative intracranial tumor volume could enhance the prognostic value of the lung-specific GPA model based on two independent cohorts (13).

The last decade saw huge progresses in NSCLC treatment by appreciating molecular characterization of the tumor and druggable targets. The therapeutic approaches for NSCLC have therefore changed since the milestone study Iressa Pan-Asia Study (IPASS) was published in 2009 (24). Nevertheless, the classical GPA, RPA, and SIR models were all reported prior to the ready availability of driver genes identification mutational status and development of druggable targets in NSCLC. Recently, several studies have addressed the favorable prognostic role of activating mutation/rearrangement status determination in BM from NSCLC (25, 26). Median OS has almost doubled for *EGFR/ALK+* NSCLC brain metastases patients compared to wild-type patients (26). As a result, Sperduto et al. revised their original disease specific-GPA scale to Lung-molGPA, which improved the prognostic ability over the RTOG RPA and the original disease specific-GPA by incorporating the impact of *EGFR* and *ALK* gene alterations on survival in patients with NSCLC and BM (27). The present study, as well as many others (27–30), have provided clearly supportive data indicating that the mutation status was an independent prognostic factor for patients with BM treated by SRS.

Anomalous energy metabolism represents a common characteristic of cancer (31). LDH, the enzyme responsible for the conversion of pyruvate to lactate during glycolysis, is known as a prognostic marker of cancer (9). Based on prospectively collected serum LDH from 7,895 patients, Wulaningsih et al. (9) demonstrated that high LDH correlated with an increased risk of death from prostate, pulmonary, colorectal, gastro-esophageal, gynecological, and hematological cancers. Our results also indicated a strong inverse association of pre-SRS serum LDH with overall survival. The underlying mechanism of LDH promotes cancer progression might related to its prominent role for basal autophagy and cancer cell proliferation (32). As a key enzyme involved in cancer metabolism, LDH also allows neoplastic cells to suppress and evade the immune system by altering the tumor microenvironment (33).

Our results did not indicate the presence of a definite correlation between OS and the number of BM or extracranial metastases, which were included in the GPA, RAP, and SIR scores. Regarding the number of metastases, the prospective JLGK 0901 study also suggested that SRS treatment outcomes in patients with five to ten brain metastases were non-inferior to outcomes in patients with two to four brain metastases (1). A case-matched study also found that OS differences between BM numbers of one to four and greater than five was only 0.9 months, which was statistically significant but clinically meaningless (34). We propose that the total volume, rather than the total number of BM is a superior prognostic factor. There was only a slight trend for worse OS for uncontrolled systematic disease (*P* = 0.12) in our cohort. This may be a result of more patients receiving systemic treatments in recent years and the availability of more effective agents, especially for Asian patients who have a higher probability of EGFR mutation (35, 36). Additionally, we did not include patients treated by adjuvant SRS following surgical resection in our cohort because we only focused on patients who received radical SRS in the present study. The prognostic value of number, diameter, and volume of brain metastases lesions might differ between adjuvant SRS and radical SRS. Thus, this nomogram may not be applicable to BM patients who have received prior surgical resection.

Potential must be appreciated in the present study. First, it was a single institutional retrospective study carrying the caveats of such studies. Although our training cohort-based nomogram was validated internally, an external validation based on multi-institutional data is needed. Secondly, the primary cancer in this cohort included mostly NSCLC patients (268, 73.9%) and a relatively small number of patients with breast cancer (38 patients), digestive system cancer (23 patients), and other cancer types (27 patients). Thus, the applicability of this nomogram for the prognostic evaluation of BM from cancer other than NSCLC should be used with caution.

In conclusion, we developed and validated a robust prognostic nomogram for patients with BM after radical SRS by integrating a panel of independent surrogate markers. In the context of targeted therapy, the established nomogram incorporating molecular biological (driver gene mutations), and radiation biological (total irradiated volume *vs* maximum tumor volume *vs* number of metastatic sites) insights, contributes to a more precise risk assessment and personalized surveillance program. However, the developed nomogram warrants further investigation in external or large-scale multi-center cohorts.

## Data Availability Statement

The raw data supporting the conclusions of this article will be made available by the authors, without undue reservation.

## Ethics Statement

The studies involving human participants were reviewed and approved by the Ethics Committee of Guangdong Sanjiu Brain Hospital. Written informed consent for participation was not required for this study in accordance with the national legislation and the institutional requirements.

## Author Contributions

Conceptualization: CZ, LW, CS, and LC. Methodology: LW, MYL, ZZ, JZ, HL, and JL. Formal analysis and investigation: LW, ZZ, CR, JW, ML, LZ, TW, and DZ. Writing—original draft preparation: LW, CZ, and CS. Writing—review and editing: CZ, GD, F-MK, LHC, and LC. Supervision: LW, CZ, F-MK, LHC, and LC. All authors contributed to the article and approved the submitted version.

## Funding

This work was supported by the Natural Science Foundation of Guangdong Province (No. 2019A1515011943), China Postdoctoral Science Foundation (No. 2019M662974) and Science and Technology Program of Guangzhou (No. 202002030445, No. 202002030086), and Medical Scientific Research Foundation of Guangdong Province (No. A2020505, No. A2020499, No. B2021203, No. B2021139). The funders had no role in study design, data collection and analysis, decision to publish or preparation of the manuscript.

## Conflict of Interest

The authors declare that the research was conducted in the absence of any commercial or financial relationships that could be construed as a potential conflict of interest.

## Supplementary Material

Supplementary Figure 1 Flow chart of the present study.

Supplementary Figure 2 An example of volumetric parameters of a female patient with two metastatic lesions in the brain.

Supplementary Figure 3 Receiver operating characteristic curve (ROC) curve for 1-year survival by cumulative tumor volume with the optimal cutoff point.
