# Supplementary figures and images for: Individualized Nomogram for Predicting Survival in Patients with Brain Metastases After Stereotactic Radiosurgery Utilizing Driver Gene Mutations and Volumetric Surrogates
Source: Front Oncol. 2021 May 13;11:659538. doi: 10.3389/fonc.2021.659538 (PMC8158152; doi:10.3389/fonc.2021.659538)

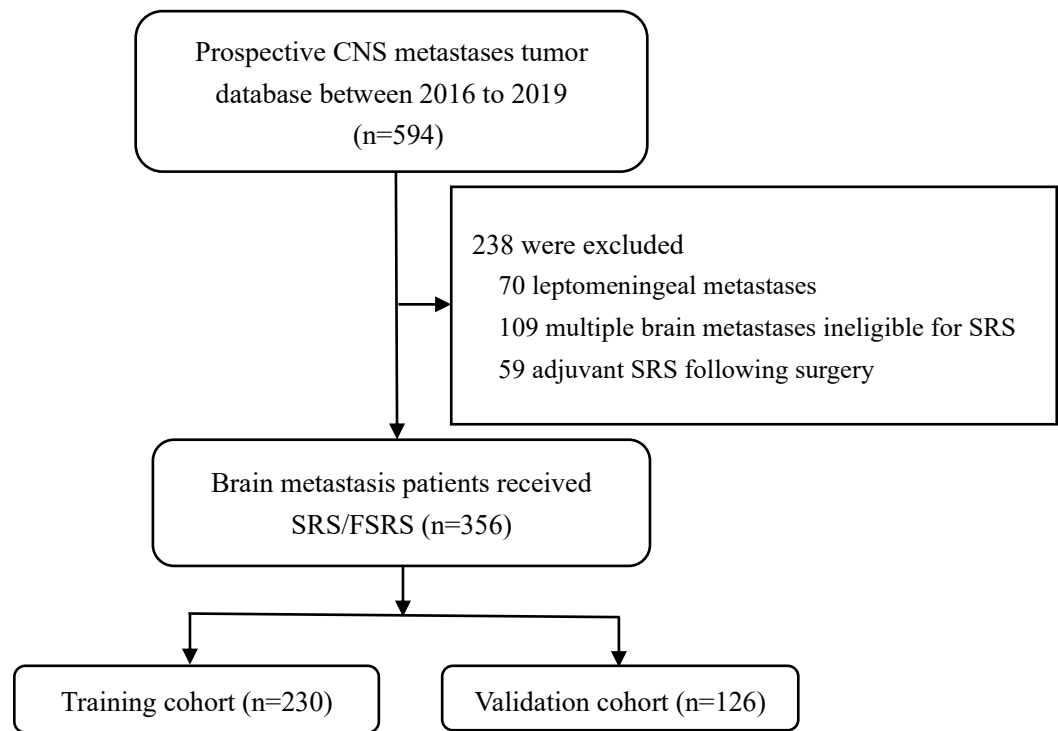

Supplement: Supplementary Figure 1 — Flow chart of the present study. [file DataSheet_1.pdf]

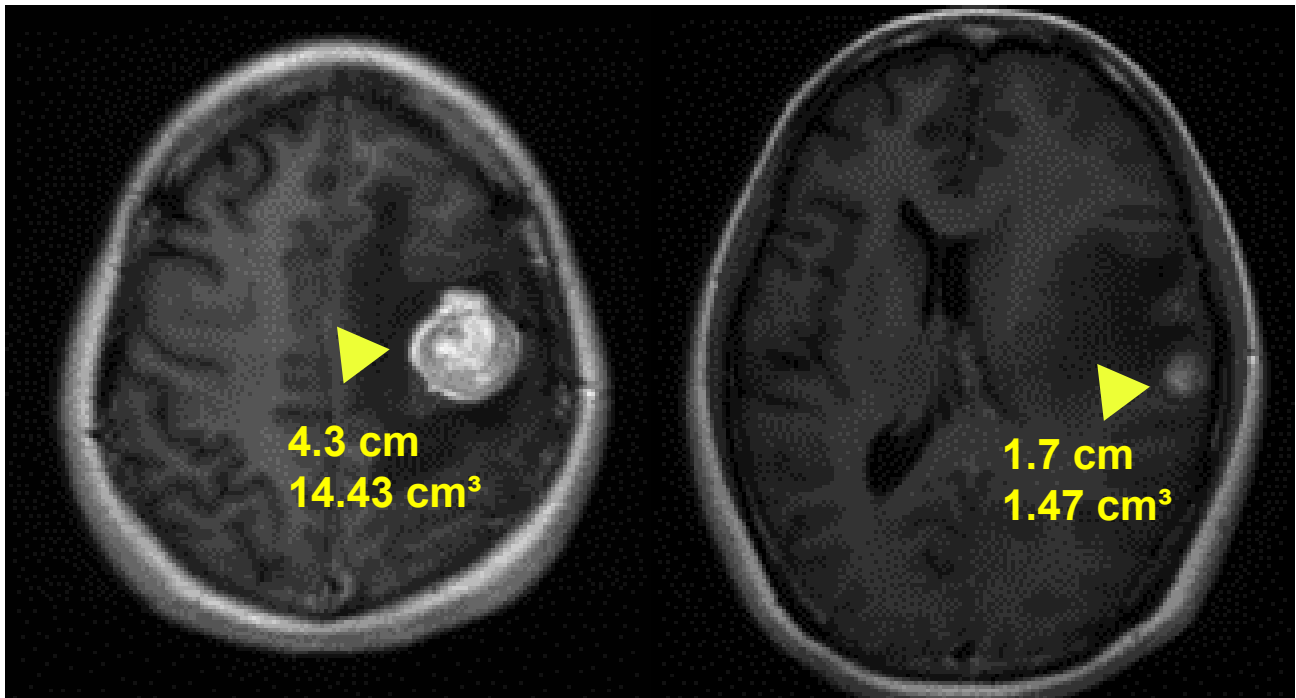

Supplement: Supplementary Figure 2 — An example of volumetric parameters of a female patient with two metastatic lesions in the brain. [file DataSheet_2.pdf]

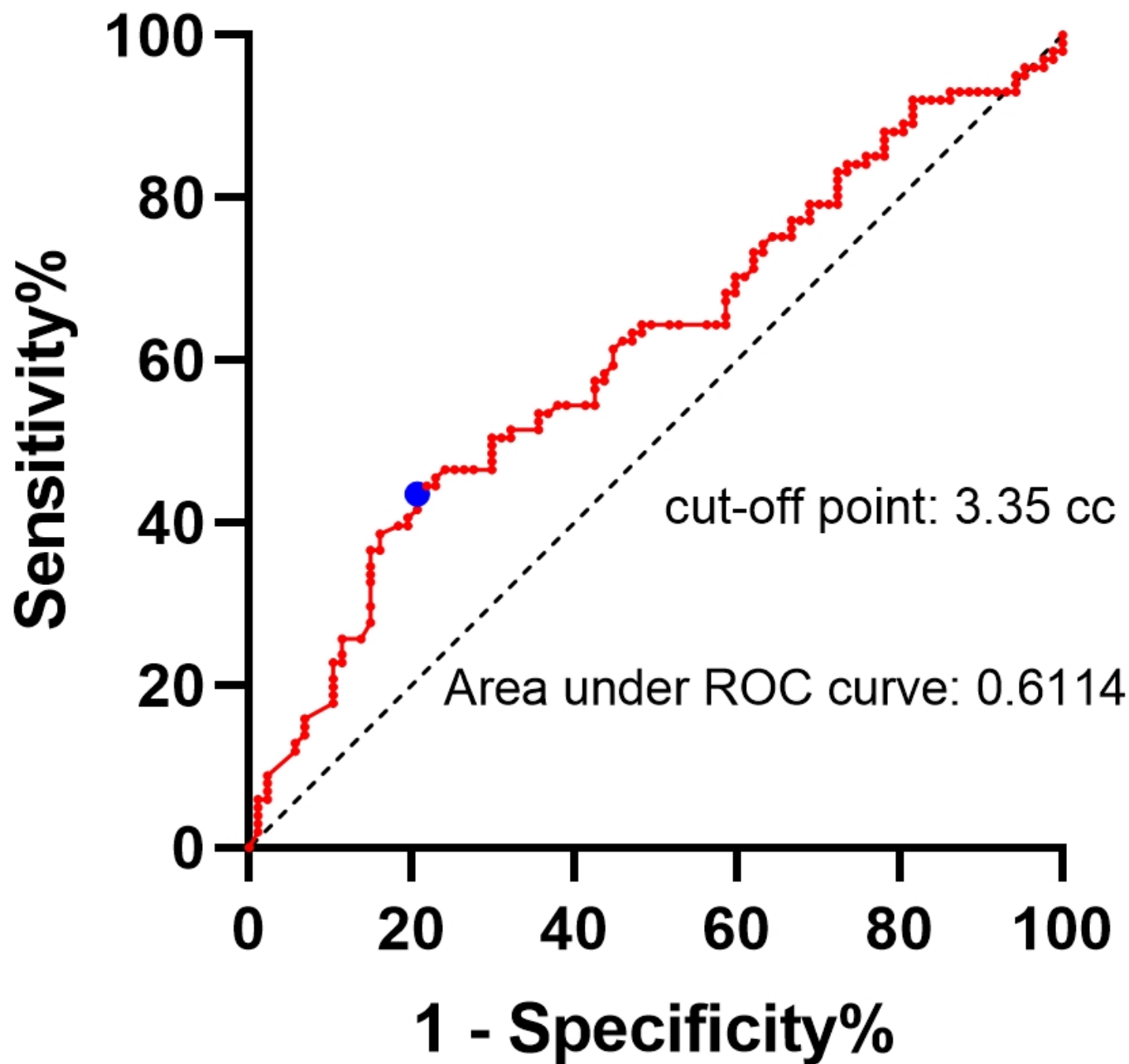

Supplement: Supplementary Figure 3 — Receiver operating characteristic curve (ROC) curve for 1-year survival by cumulative tumor volume with the optimal cutoff point. [file DataSheet_3.pdf]
